# Larvicidal activity of plants from Myrtaceae against *Aedes aegypti* L. and *Simulium pertinax* Kollar (Diptera)

**DOI:** 10.1590/0037-8682-0092-2020

**Published:** 2020-12-21

**Authors:** Vanessa Cristine de Souza Carneiro, Luana Braz de Lucena, Ronaldo Figueiró, Cristiane Pimentel Victório

**Affiliations:** 1 Fundação Centro Universitário Estadual da Zona Oeste, Programa de Mestrado em Ciência e Tecnologia Ambiental, Rio de Janeiro, RJ, Brasil.; 2 Universidade Castelo Branco, Centro Universitário de Volta Redonda, Volta Redonda, RJ, Brasil.; 3 Fundação Centro Universitário Estadual da Zona Oeste, Laboratório de Pesquisa em Biotecnologia Ambiental, Rio de Janeiro, RJ, Brasil.

**Keywords:** Alternative chemical control, Plant extracts, Larvicide, Mosquitocide, Plant natural products, Sandy coastal plains

## Abstract

**INTRODUCTION:**

Despite their widespread usage, synthetic insecticides and larvicides are harmful for controlling disease-causing mosquitoes owing to the development of resistance. The leaves of *Eugenia astringens*, *Myrrhinium atropurpureum*, and *Neomitranthes obscura* were collected from Marambaia and Grumari restingas. The safety and larvicidal efficacy of their extracts were tested against *Aedes (Stegomyia) aegypti* L. and *Simulium (Chirostilbia) pertinax* Kollar.

**METHODS:**

The dry leaves were subjected to static maceration extraction using 90% methanol. *A. aegypti* and *S. pertinax* larvae were exposed to 7.5, 12.5, and 25.0 µL/mL of the extracts (n= 30). The larvicidal activity after 24 h and 48 h, and the mortality, were determined. The median lethal concentration (CL_50_) was estimated by a Finney's probit model.

**RESULTS:**

*M. atropurpureum* and *E. astringens* extracts exhibited the strongest larvicidal effects against *A. aegypti*. *M. atropurpureum* extracts (25 µL/mL) caused mortalities of over 50% and 100% after 24 h and 48 h, respectively (CL_50_ = 11.10 and 9.68 ppm, respectively). *E. astringens* extracts (25 µL/mL) caused mortalities of 50% and 63.33% after 24 h and 48 h, respectively. High concentrations of *N. obscura* extracts induced a maximum mortality of 46.66% in *A. aegypti* larvae after 48 h (CL_50_= 25 ppm). The larvae of *S. pertinax* showed 100% mortality following exposure to all the plant extracts at all the tested concentrations after 24 h.

**CONCLUSIONS:**

The extracts of *M. atropurpuerum* exhibited the strongest larvicidal activity against *A. aegypti*. The larvae of *S. pertinax* were sensitive to all the extracts at all the tested concentrations.

## INTRODUCTION

Dengue, yellow fever, zika, chikungunya, filariasis, onchocerciasis, and mansonellosis are some of the diseases transmitted by mosquito vectors. *Aedes aegypti* L. transmits different viruses that cause diseases throughout the Americas, Southeast Asia, and Western Europe. These vectors are also responsible for causing disorders that arise indirectly from viral infections, including microcephaly and Guillain-Barré syndrome. Globally, a million people are affected by diseases transmitted by *A. aegypti* on an annual basis, and this is most pronounced in summer^1^. The hematophagous species, *Simulium pertinax* Kollar, also known as black flies or “borrachudos” in Brazil, is an pest of considerable concern for both humans and domestic animals. Only a limited number of studies on Simuliidae can be found in the literature. However, the simuliids proliferate uncontrollably in some parts of the world, and cause a profound deterioration in the quality of life by acting as vectors of pathogenic organisms, and as hematophagous pests of humans and domestic animals^2^. However, some studies have reported that *S. pertinax* acts as a bioindicator of moderately impacted streams^3^.

Mechanical control is the most common means of controlling mosquito vectors, and comprises the elimination of the vector and its breeding sites, or the reduction of mosquito-human contact. Another strategy involves biological control, which is based on the use of predators or pathogens that potentially reduce the vector population^4^. Apart from these strategies, synthetically-derived chemicals are also used to kill the larval and adult stages of mosquito vectors^5^. For several decades, the use of synthetic insecticides has been a widespread strategy for controlling disease-causing mosquitoes. However, the prolonged and indiscriminate use of synthetic insecticides has resulted in the development of resistance to these substances^6^. Insecticide resistance is currently a major threat to the control of insects, including *A. aegypti* ^7^ and *S. pertinax* ^8^. Apart from the development of resistant mosquito populations, the use of such insecticides increases environmental pollution and the risk of toxicity in human beings, which are issues of increasing global concern. This necessitates the development of natural substances that act at different stages of the life cycle of insects, which comprises the egg, larva, pupa, and adult stages. Natural agents are less toxic and safer than synthetic insecticides, and offer an alternative strategy for vector control^9^. Some studies have demonstrated that various species of plants possess larvicidal and insecticidal potential. It is estimated that approximately 479 articles were published between 1968 and 2016, which evaluated the activity of natural plant products against *A. aegypti* ^10^ ^,^ ^11^ ^,^ ^12^. Amides, quinones, and terpenoids are active larvicidal compounds, and represent the major natural substances responsible for controlling *A. aegypti* ^*10*^ ^*,*^ ^*13*^. Biolarvicides, based on mosquitocidal toxins derived from strains of *Bacillus thuringiensis* (Bt), are globally used on a large scale for their pathogenicity and specificity against the larval stages of *S.* sp.^14^.

Numerous species of plants from Myrtaceae are found in the slopes of the ombrophilous forests or Atlantic forests, Amazon rainforest, Restinga, and Cerrado regions of Brazil^14^. For instance, the preserved area of Marambaia restinga in Rio de Janeiro houses a wide diversity of plant species belonging to this family, and are present in seven of the eleven plant formations defined in the restingas^15^ ^,^ ^16^ ^,^ ^17^. Numerous species are also found in Grumari restinga, which, despite being located in an urban area and being subject to anthropic activity, still houses remnants of the restinga^18^. Several species of plants from Myrtaceae are used for medicinal purposes, including the treatment of gastrointestinal disorders, hemorrhagic conditions, and infectious diseases, and it is thought that the underlying mechanism of action is related to the astringent properties of the plants. The most commonly used plant parts are the leaves, bark, and fruits^19^. However, there is a scarcity of studies on their use and efficacy against insect vectors^20^ ^,^ ^21^.

In this study, we therefore aimed to investigate the larvicidal activity of different leaf extracts of plants from the Myrtaceae family as natural alternatives to chemical insecticides. The use of plants from the Myrtaceae family is further justified by the diversity of species found in the restinga environments of Rio de Janeiro.

## METHODS

### Restinga areas and collection of plant material

**FIGURE 1:**
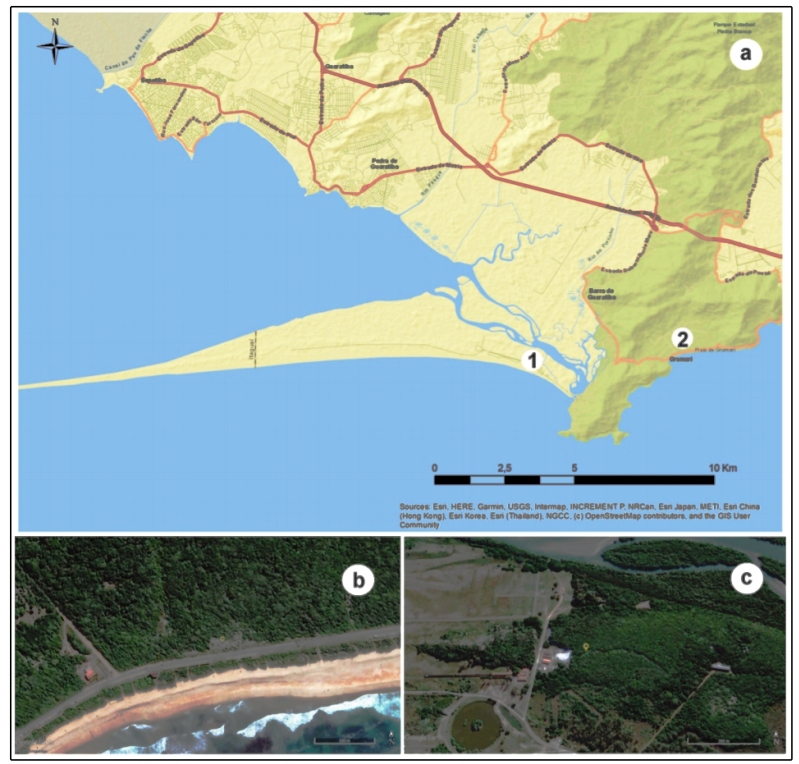
(a) Collection areas in the West Zone of the restingas in Rio de Janeiro: 1. Marambaia and 2. Grumari. The collection points at Grumari (b) and Marambaia (c) are indicated in yellow. Program ArcMap 10.6.

The study was conducted in the Laboratory of Environmental Biotechnology, Fundação Centro Universitário Estadual da Zona Oeste (UEZO), Rio de Janeiro, Brazil. The research was conducted by students pursuing their Masters degree. The extracts were processed from the leaves of 3 species of plants of the Myrtaceae family that were collected from the restingas ecosystem in the West Zone of Rio de Janeiro. These plants included *Eugenia astringens* Cambess (*syn*. *E. rotundifolia* Casar., *E. umbelliflora* O.Berg) and *Myrrhinium atropurpureum* Schott, which were collected from Grumari restinga (23º02'53.3", 23º03'10"S; 43º31'45.1", 43º32'30"W), and *Neomitranthes obscura* (DC.) N. Silveira growing in the preserved area of the Marambaia restinga, “Line 2” (23°04'S, 44°00'W; 23°02'S, 44°34'W) (Figure 1). The leaves were collected between the months of February and March of 2017. The plants were identified by the taxonomist, Marcelo da Costa Souza, and were deposited in the herbarium collections of the Rio de Janeiro Botanical Garden (RB), Universidade Federal do Rio de Janeiro (RBR), and Universidade Federal do Estado do Rio de Janeiro (HUNI). The deposition numbers were: *M. atropurpureum* - RB415731, *N. obscura* - RBR12592, and *E. astringens* - HUNI650. The collected materials were dried in an environment with constant air circulation at 28^o^C±2^o^C for a period of approximately one month. The permission for sample collection was obtained from the Ministry of Environment (SISBIO/ICMBio) under approval number 37376-2. 

### Preparation of hydroalcoholic plant extracts

The dried leaves of *E. astringens* (44 g), *M. atropurpureum* (58 g), and *N. obscura* (34 g) were shredded and placed in 500 mL beakers, into which 450 mL of a solution of 90% methanol:distilled water was added. The extraction was performed by static maceration for 10 days at 28^o^C±2^o^C, and the setup was protected from light during this process. The extracts were subsequently filtered using a paper filter. The solvent was evaporated on a rotary evaporator at 50°C and the aqueous residues were removed by lyophilization.

###  Bioassay for determining larvicidal activity against *A. aegypti*

In order to determine the larvicidal activity of the extracts against *A. aegypti* by bioassays*,* the eggs of *A. aegypti* were obtained from *Núcleo Operacional Sentinela de Mosquitos Vetores* (NOSMOVE/FIOCRUZ) and placed in two tulle-covered trays containing 750 mL of filtered water. After hatching, which occurred within 24 h, the larvae were fed on a yeast-based diet until they reached the third instar stage, determined by the head/body size ratio.

For the biological assays, the extracts were prepared at three concentrations of 25, 12.5, and 7.5 µL/mL, corresponding to 500, 250, and 150 µL/L, respectively. These extracts were prepared from a stock solution containing 150 mg of each extract, dissolved in 3 mL dimethyl sulfoxide (DMSO). The solutions were subsequently placed in an ultrasound bath for 1 h. The tests were performed in 200 mL plastic cups containing 20 mL of distilled water. Each treatment was repeated thrice using ten experimental setups comprising 10 cups with 10 third instar larvae each (n= 30). Water and DMSO were used as the controls. The results were recorded after 24 h and 48 h, by counting the dead larvae that did not react to the mechanical stimuli provided by light and tweezers.

###  Larvicidal bioassay against *S. pertinax*

**FIGURE 2:**
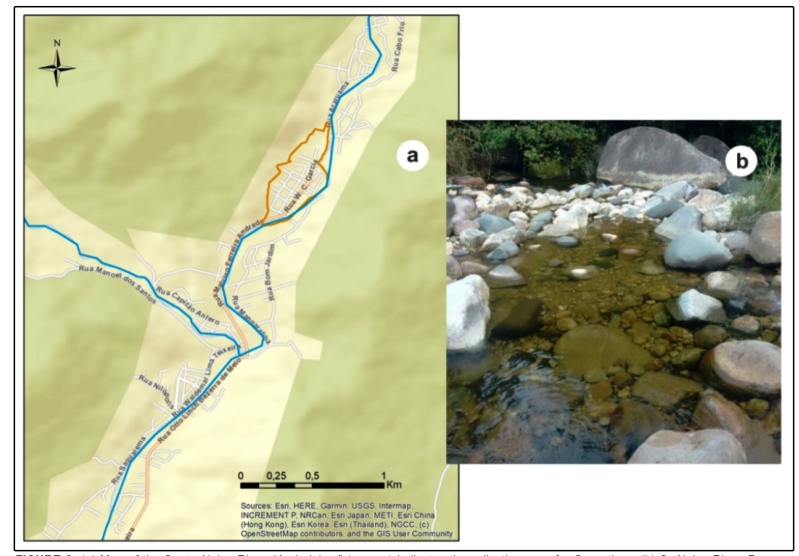
(a) Map of the Santo Aleixo River, “Andorinhas” (orange) indicates the collection area for *S. pertinax*. (b) S. Aleixo River. Program ArcMap 10.6.

In the present study, the samples were collected from the Santo Aleixo River (Figure 2). The Santo Aleixo River is in the neighborhood of Andorinhas in Santo Aleixo District under the jurisdiction of Magé municipality, located in the biogeographic province of Serra do Mar, Serra dos Órgãos region, in Rio de Janeiro (22°39'10'' S, 43°02'26'' W; S 22^o^32', W 43^o^02'. The Santo Aleixo River is part of the Paraíba do Sul River Basin that falls within the tropical Atlantic morphoclimatic region. The climatic conditions of this area ranges from hot and sub-hot to super humid, and comprise an intermediate sub-drought period.

**FIGURE 3:**
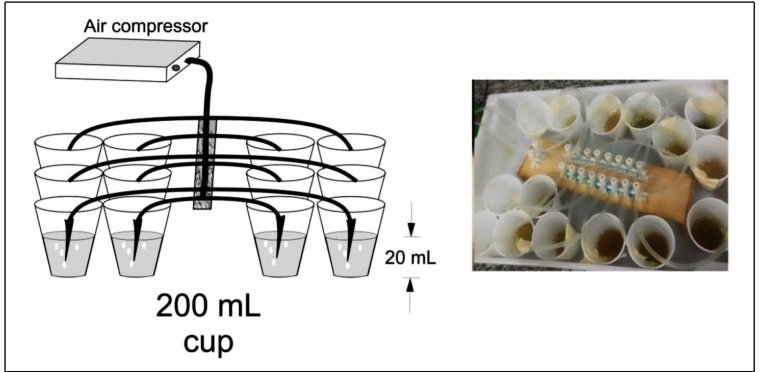
Equipment used for transporting the simuliid larvae and performing bioassays with the plant extracts.

The samples were collected from each site using the transect method in which all the larvae present within a 5 m stretch of the river were collected. The larvae were collected from rocky substrates and the shafts of the dams along the watercourse, and the larvae that adhered to the substrate were removed with tweezers. The collected materials were stored in covered plastic pots (200 mL) containing 20 mL distilled water, which were placed in a styrofoam box. The pots were aerated by flowing air from an air compressor for simulating running water. Biological ice was used to maintain temperature at 20^o^C±2^o^C and to ensure larval survival for the subsequent bioassays. The covers on the plastic pots were perforated to allow the insertion of a plastic tip connected to a silicone hose that permitted the aeration of the pots. This equipment was used for transporting the samples and for performing the bioassays (Figure 3)*.* Simuliids are aquatic insects found in flowing watercourses (rheophilic) with different volumes of water. They live in environments with high dissolved oxygen content and feed on finely dissolved organic particles suspended in water^22^ ^,^ ^23^ ^,^ ^24^. The collected larvae were then transported to the laboratory after approximately two hours of collection, and the last instar larvae were selected using a stereoscope for performing bioassays for evaluating the larvicidal potential of the extracts at different concentrations. The same aforedescribed equipment was used for this bioassay, with the exception that 200 mL cups were used instead of plastic pots, as depicted in Figure 3. Water and DMSO were used as the controls. Three concentrations of the extract (25, 12.5, and 7.5 µL/mL) were used for the biological assays. The extracts were prepared from a stock solution containing 150 mg of each of the extracts that were separately dissolved in 3 mL DMSO and placed in an ultrasound bath for 1 h. The larvicidal activity of the extracts were evaluated in triplicate using ten cups (n= 30), according to a method described previously^4^. Each of the cups contained the leaf extracts that were diluted in 20 mL distilled water. Each of the cups contained 10 simuliid larvae, and the number of dead larvae were counted at 24 h and 48 h.

### Statistical analyses

All the data were initially analyzed by Lilliefors normality tests, and subsequently by the Kruskal-Wallis test if the data distributions were not normal, for determining the statistical differences between the control group and the three treatment groups that were administered different plant extracts at different concentrations, at a significance level of p< 0.05. The percentage mortality (%) and median mortality were also determined using a Finney’s probit model for estimating the median lethal concentration (CL_50_)^25^ with 95% confidence limits.

## RESULTS

###  Larvicidal activity against *A. aegypti*

Only the methanolic extracts of *M. atropurpureum* and *E. astringens*, at concentrations of 7.5, 12.5, and 25 µL/mL, showed larvicidal activity after 24 h. It was also evident that DMSO had no impact on larval mortality, as depicted in Table 1. The extracts of *M. atropurpureum* exhibited the strongest larvicidal activity at a concentration of 25 µL/mL. This resulted in a total larval mortality of 100% in all the repetitions. At a lower concentration of 12.5 µL/mL, larval mortality was higher than 50%. The extract of *E. astringens* showed promising results at a concentration of 25 µL/mL, with 50% larval mortality after 24 h of treatment, as depicted in Table 1.

**TABLE 1:** Larvicidal activity of methanolic extracts (90%) of Myrtaceae plant species against *A. aegypti* after 24 hours (comparison between treatment and control triplicates).

Samples	Concentration	P-value	Mortality	Mortality Median	CL_50_ (ppm)	95% confidence
	(µL/mL)		(%)	(Q1, Q3) (%)		interval
*Neomitranthes obscura*	7.5	0.6472	3.33±4.71	10(5,15)		
	12.5	0.6472	3.33±4.71	0(0,0)	n.e.	n.e.
	25.0	0.4805	1.00±14.14	50(40,55)		
*Myrrhinium atropurpureum*	7.5	0.2014	20.0±21.60	20(10,45)		
	12.5	0.005691*	66.66±9.42	70(60,75)	11.10	8.62 - 14.30
	25.0	0.001357*	100.0±0	100(100,100)		
*Eugenia astringens*	7.5	0.36	6.66±4.71	40(20,50)		
	12.5	0.04131	30.0±14.14	40(40,50)	23.58	14.64 - 37.95
	25.0	0.01465*	50.0±21.60	80(55,80)		
Water (control)	-	1.0	0±0	0(0,0)	-	-
DMSO (control)	7.5	1.0	0±0	0(0,0)	-	-
	12.5	0.541	6.66±9.42	0(0,10)		
	25.0	1.0	0±0	0(0,0)		

*Significant differences, p< 0.05, according to Kruskal-Wallis test. CL_50 -_ median lethal dose. n.e. - no effectiveness after 24 h. (-) not calculated.

The toxicity of the extracts was also evaluated by Finney’s probit analysis method^25^, and the results are expressed in terms of CL_50_. The CL_50_ values of the methanolic extracts of *M. atropurpureum* and *E. astringens*, determined using third instar larvae, were 11.10 ppm and 23.58 ppm, respectively. The CL_50_ values of the extracts of *N. obscura* could not be calculated owing to insufficient efficacy after 24 h of treatment.

The larvicidal effects of the methanolic extracts of the leaves of *M. atropurpureum* and *E. astringens*, at concentrations of 12.5 and 25 µL/mL, and that of the *N. obscura* extract at a concentration of 25 µL/mL (46.66% mortality), were verified after 48 h of treatment (Table 2).

 The larvicidial effect of *N. obscura* was evident at higher concentrations, and caused a maximum mortality of 46.6% after 48 h of treatment. However, the other plant species showed no significant differences with respect to the duration of exposure. The CL_50_ values of the leaf extract of *M. atropurpureum* and methanolic extract of *E. astringens* after 48 h were 9.68 ppm and 15.32 ppm, respectively*.* The CL_50_ value of the leaf extract of *N. obscura* was 25 ppm, as depicted in Table 2. The methanolic extracts of *M. atropurpureum* showed maximum larvicidal activity, resulting in 100% mortality.

**TABLE 2:** Larvicidal activity of methanolic extracts (90%) of Myrtaceae plant species against *A. aegypti* after 48 hours (comparison between treatment and control triplicates).

Samples	Concentration	P-value	Mortality	Mortality Median	CL_50_ (ppm)	95% confidence
	(µL/mL)		(%)	(Q1, Q3) (%)		interval
*Neomitranthes obscura*	7,5	0.4541	10±8.16	10(5,15)		
	12.5	1.0	0±0	0(0,0)	25.0	15.54 - 40.21
	25.0	0.04728*	46.66±12.47	50(40,55)		
*Myrrhinium atropurpureu*m	7.5	0.1966	30±29.47	20(10,45)		
	12.5	0.01092*	66.66±12.47	70(60,75)	9.68	7.42 - 12.62
	25	0.001128*	100±0	100(100,100)		
*Eugenia astringen*s	7.5	0.1661	33,33±24,94	40(20,50)		
	12.5	0.04728*	46.66±9.42	40(40,50)	15.32	7.41 - 31.66
	25.0	0.01422*	63.33±23.57	80(55,80)		
Water (control)	-	1.0	0±0	0(0,0)	-	-
DMSO (control)	7.5	1.0	0±0	0(0,0)		
	12.5	0.6805	6.66±9.42	0(0,10)	-	-
	25.0	10.0	0±0	0(0,0)		

*Significant differences, p< 0.05, according to Kruskal-Wallis test. (-) not calculated.

###  Larvicidal activity against *S. pertinax*

After the 24 h of treatment, 100% mortality was observed in the larvae of S. pertinax in all the treatment groups, while DMSO had no impact on larval mortality. In fact, all the larvae in the control groups remained alive after the treatment period. This strongly suggested that these plant extracts had larvicidal activity. However, as the mortality rate was 100%, the CL_50_ value could not be determined.

This study is the first to elucidate the larvicidal activity of plants of the Myrtaceae family against simuliid larvae. However, it was not possible to determine the precise concentration of the extract at which the simuliid larvae were killed. Nonetheless, this study suggested the high sensitivity of S. pertinax to the plant extracts studied herein.

## DISCUSSION

The larval stage represents the most delicate stage in the life cycle of insect vectors. Therefore, efforts to control these vectors are usually directed at the larval stage for disrupting their biological cycle by different strategies. The most commonly used forms of vector control include mechanical, biological, and chemical strategies. Several studies have been performed on plant extracts with the aim of developing a method for the biological control of vectors, that would have minimal environmental impact and adverse effects on the local fauna and flora. Myrtaceae, Lamiaceae, and Rutaceae are the most frequently studied plant families, owing to their larvicidal and insecticidal properties^26^. In a previous study, the larvicidal property of *Eugenia candolleana* was evaluated using the essential oils extracted from hydrodistilled leaves. The results proved to be highly satisfactory against the larval stage of *A. aegytpi*, with a mortality of 100% within 24 h of treatment. This observation is in agreement with that of the present study, in which the larvicidal activity of the leaf extracts of *M. atropurpureum* was determined in terms of percentage mortality. The results of our study demonstrated that the leaf extracts of *M. atropurpureum* caused 100% mortality after 24 h of treatment. It has been reported that the essential oils extracted from the leaves of *Pimenta pseudocaryophyllus* have larvicidal activity against *A. aegypti* (CL_50_ = 44.09 ppm)^27^. Numerous studies have reported the larvicidal activity of essential oils extracted from various plants. It has been demonstrated that the polar extracts of the leaves and fruits of *Callistemon citrinus* have larvicidal activity, and induce various malformations in the fourth instar larvae of *A. aegypti* following exposure to the extract^28^.

The results of our study are interesting as the determinant of larval toxicity of a substance or phytocomplex is the efficacy, even at low concentrations, among other properties. Compared to the concentration used in other studies, we treated the larvae with the extracts at a relatively low concentration of 25 µL/mL^28^ ^,^ ^29^. Numerous studies have evaluated the toxicity of natural products in terms of the CL_50_ value, concentration of the sample that is responsible for inducing the concerned effect in 50% of the test organisms. A low CL_50_ value indicates a higher toxicity of the sample. The results of this study are promising owing to the fact that the CL_50_ value of the methanolic extracts of *M. atropurpureum* after 24 h was 11.10 ppm. The values obtained herein are comparable to those obtained by Govindarajan and Karuppannan^30^ who reported that the maximum larvicidal activity of the methanolic extracts of *Eclipta alba* (Asteraceae) was observed at 127.64 ppm. The extract prepared herein can be considered to be effective at relatively low concentrations. Several authors, including Komalamisra and coworkers^31^, suggest that a natural product with a CL_50_ value of 50 mg/L is active, and if the CL_50_ value varies between 50 mg/L and 100 mg/L, the natural product is said to be moderately active. The CL_50_ values obtained in our study were below 50 mg/L. The leaf extracts of *M. atropurpureum* and *E. astringens* caused 30 and 33.33% mortality, respectively, after 24 h, at the lowest concentration of 7.5 µL/mL. These data had very high values of standard deviation, which were higher than the mean values in some cases. These results suggested that more bioassays are necessary for proving the efficiency of the extracts at certain concentrations, although the sampling performed for the larvicidal assays was satisfactory. However, treatment with 25 µL/mL methanolic extracts of *M. atropurpureum* caused 100% mortality in the *A. aegypti* larvae, indicating the larvicidal efficacy of this extract. In terms of the duration for which the treatments were conducted, the highest concentrations of *N. obscura* and *E. astringens* tested herein showed a higher activity compared to that of the other treatments, after 24 and 48 h. Nonetheless, the extracts of *N. obscura* were found to be ineffective against the larval stages. These results suggested that the duration of treatment should be 24 h.

There are few studies on the larvicidal activity of natural products against simuliid larvae. Some studies have been conducted in India, using the roots and leaves of *Ocimum gratissimum*, *Azadirachta indica* (popularly known as “neem”), *Pterocarpus santalinoides*, and *Pistia hyptis* ^32^. The results of our study indicated that all the plant extracts tested herein had larvicidal activity; however, the CL_50_ values could not be determined owing to a mortality rate of 100%. 

Previous studies have reported the phytochemical data pertaining to the essential oils present in the leaves of the species growing in Grumari restinga. The studies demonstrated that sesquiterpenes are the main constituents of the leaves of *N. obscura*, while pinene monoterpenes are the major volatile compounds in the leaves of *E. astringens* and *M. atropurpureum* ^16^ ^,^ ^33^. Studies on the polar extracts of the leaves demonstrated the presence of phoroglucinols (eugenial C, eugenial D, and eugenial E) and pentacyclic triterpenes (α-amirin, ꞵ-amirin, and betulinic acid) in *E. astringens* ^34^ ^,^ ^35^. On the other hand, the flavonoid, quercitrin, is the main component of *N. obsucura* (data not shown).

The methanolic extract of *M. atroporpureum* was highly lethal to the larval stage of *A. aegypti* at a concentration of 25 µL/mL, resulting in 100% mortality after 24 h (CL_50_ = 11.10 ppm), and its larvicidal activity was higher than that of the other extracts tested herein. This extract showed more promise for the control of mosquito larvae. Our study further demonstrated that the 24 h treatment period is sufficient for efficacy. The extracts of *E. astringens* also showed larvicidal activity, with a larval mortality rate of 50% (CL_50_ = 23.58 ppm). After 48 h of treatment, the CL_50_ values of the leaf extracts of *E. astringens* and *M. atroporpureum* were 15.32 ppm and 9.68 ppm, respectively. The results of this study demonstrated that the methanolic extracts of *M. atropurpureum, E. astringens*, and *N. obscura* have significant larvicidal potential against the larval stages of Simuliidae; however, further studies are necessary for determining the lethal concentrations (CL_50_) of each of the leaf extracts.

This work contributes to the literature on the vegetation growing in the restinga areas of Rio de Janiero, and increases the probability of finding new natural compounds with enhanced larvicidal activity that can be used for biological vector control. Further studies are necessary for isolating and identifying the active phytochemicals present in the extracts of *E. astringens*, *M. atropurpureum*, and *N. obscura*. It is also necessary to perform bioassays for identifying the compounds that are responsible for larval mortality and for determining whether these compounds act synergistically. These natural products can be used in the future as a tool for the biological control of *A. aegypti* and *S. pertinax* larvae.
